# DNA G-quadruplex-stabilizing metal complexes as anticancer drugs

**DOI:** 10.1007/s00775-022-01973-0

**Published:** 2022-12-02

**Authors:** Jaccoline Zegers, Maartje Peters, Bauke Albada

**Affiliations:** Laboratory of Organic Chemistry, Wageningen University and Research, Stippeneng 4, 6708 WE Wageningen, The Netherlands

**Keywords:** Guanine tetrads, Oncology, Bioinorganic chemistry, Metallodrugs

## Abstract

Guanine quadruplexes (G4s) are important targets for cancer treatments as their stabilization has been associated with a reduction of telomere ends or a lower oncogene expression. Although less abundant than purely organic ligands, metal complexes have shown remarkable abilities to stabilize G4s, and a wide variety of techniques have been used to characterize the interaction between ligands and G4s. However, improper alignment between the large variety of experimental techniques and biological activities can lead to improper identification of top candidates, which hampers progress of this important class of G4 stabilizers. To address this, we first review the different techniques for their strengths and weaknesses to determine the interaction of the complexes with G4s, and provide a checklist to guide future developments towards comparable data. Then, we surveyed 74 metal-based ligands for G4s that have been characterized to the in vitro level. Of these complexes, we assessed which methods were used to characterize their G4-stabilizing capacity, their selectivity for G4s over double-stranded DNA (dsDNA), and how this correlated to bioactivity data. For the biological activity data, we compared activities of the G4-stabilizing metal complexes with that of cisplatin. Lastly, we formulated guidelines for future studies on G4-stabilizing metal complexes to further enable maturation of this field.

**Graphical abstract:**

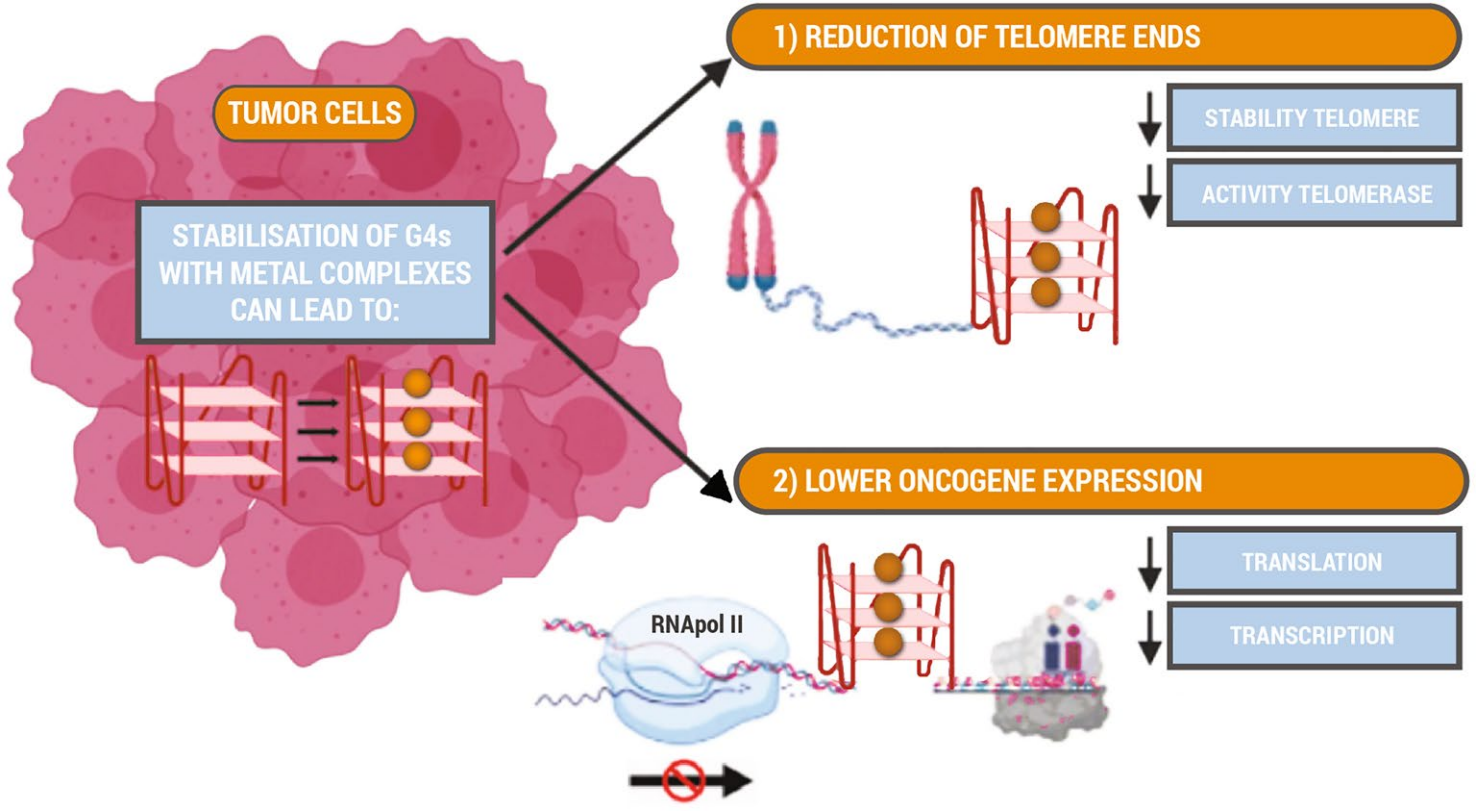

## Introduction

Cisplatin was the first and only metal-based drug that was approved by the FDA as chemotherapeutic agent for the treatment of cancer in the clinic [1]. Despite major drawbacks of serious dose-limiting side effects associated with cisplatin, nearly 50% of all cancer treatments involve the administration of this first-and-only-in-class drug [1, 2]. In view of this unfavorable combination of high potency and substantial side effects associated to cisplatin treatments, efforts to find novel metal-based anticancer drugs increased [3]. Much of this evolved around the development of metal complexes that strongly bind to specific proteins or DNA structures associated with cancer. Interestingly, whereas cisplatin and other Pt-based drug candidates mostly bind to *i*,*i* + 2 guanine (G) bases in B-DNA [4], other forms of DNA that correlate to the occurrence of cancer can be targeted as well.

The so-called G-quadruplex DNA structure gained a lot of attention in the last 20 years as a promising target for new anticancer drugs [5]. This particular interest was caused by its abundance in the promoter regions of oncogenes but also at the telomeric ends of DNA where it controls the number of copies that can be made from the DNA strand promoter [6]. Recently, RNA guanine-rich regions emerged as potential target for anticancer drugs as well [7]; these quadruplexes are thermodynamically more stable, more compact, and less hydrated [7]. In this review, however, we will focus on DNA G-quadruplexes as they are investigated more widely.

The first section of this review describes the formation and geometries of G-quadruplexes and indicates its relevant importance in cancer treatment. For more comprehensive overviews of the roles of G4s in biology and cancer, the reader is referred to reviews dedicated to those topics (see ‘The roles of G-quadruplexes in biology and cancer’). After this, we briefly describe and evaluate the techniques that are used to study the molecular interactions of the ligands with the G-quadruplex structure. The major part of this review will focus on metal complexes specifically designed to interact with DNA G-quadruplex structures [8, 9]. These complexes are divided in the following categories: metalloporphyrins, metallophthalocyanines and metallocorroles, and complexes based on metal–salphen or metal–salen, planar metal–phenanthroline, metal–terpyridine, octahedral ruthenium, multinuclear metal assemblies, cisplatin derivatives, and lastly miscellaneous complexes that could not be assigned to a previously treated category. Evaluation of these complexes revealed that a current major challenge is finding an appropriate balance between selectivity and affinity of the metal complex for cancer-related G4s over dsDNA. Therefore, it is crucial to extensively characterize G-quadruplex/ligand interactions as a restricted selection of techniques does not provide sufficient guidance for interpretation of the biological data [10]. We noticed that a diverse array of techniques and methods have been used to study the interaction between an even wider variety of metal complexes and relevant G-quadruplexes, making comparison between the results nearly impossible. As the field is extensively researched, we felt it would be time to evaluate this, and to provide guidelines for future research in order to be able to better compare the results of the different studies as a predictor for biological performance. The enormous potential that is stored in this G-quadruplex-stabilizing complexes is then harvested more efficiently.

## Structure of G-quadruplexes

**Fig. 1:**
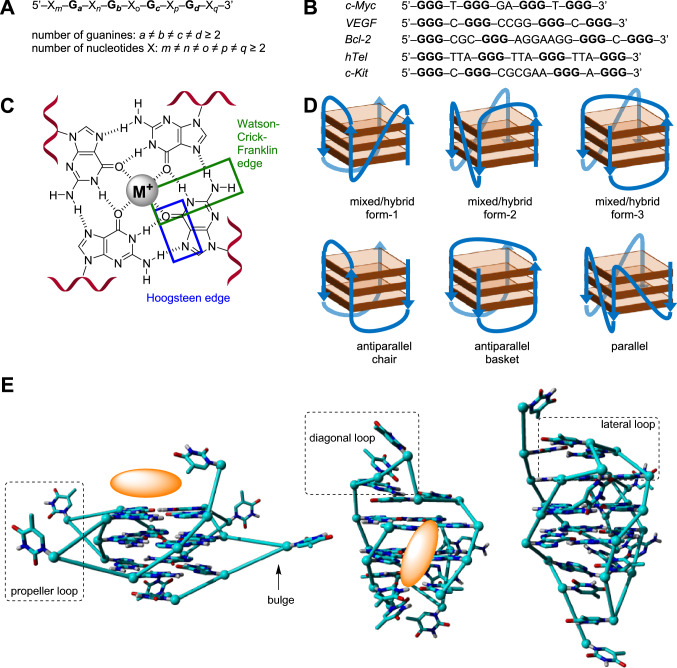
**A** Generic G-quadruplex forming sequence, the ≠ sign indicates the large variations observed in G4s. **B** Sequences of G-quadruplex forming sequences mentioned in this review. **C** Chemical drawing of a guanine tetrad, with the different hydrogen bonding interactions and the centra metal monocation that is required to stabilize the G4 biological nanostructure. Representation of a G4-DNA structure consisting of three stacked G-quartets. **D** Various conformations of G-quadruplexes that are known to occur in nature. **E** Structures of biological G4 structures resolved by NMR (left: parallel G-quadruplex [PDB-code: 2M4P]; middle: antiparallel G-quadruplex [PDB-code: 1L34], right: hybrid G-quadruplex [PDB-code: 2JPZ]). Images were generated using YASARA and based on NMR structures of which one representative structure has been displayed out of the 10 lowest energy structures that were deposited. Two common binding modes for G4-stabilizing ligands are shown by the orange sphere in the first two structures: end-on G4 stacking (left) and major groove binding (right)

G-quadruplexes can be found in guanine-rich regions of eukaryotic chromosomes [6]. A G-quadruplex can be formed by a guanine-rich strand (Fig. 1A) if stacked G-quartets (a.k.a. tetrads) are sufficiently stabilized by monovalent cations M^+^ in the center, usually Na^+^ and K^+^, and by π–π interactions (Fig. 1C) [11]. G-quartets consist of four guanine residues that can self-assemble in a square planar format through hydrogen bonding and lone pair metal–ion interactions (Fig. 1C) [12]. G-quadruplexes occur in a wide variety of topologies that arise from variations in the number of stacked guanine tetrads, the loops that connect these, and the directions by which the backbone connects the nucleotides [12] (Fig. 1D, E). In general, G-quadruplexes can be classified as antiparallel, parallel, or as hybrid when considering the direction of the strands (Fig. 1D), whereas the loops are usually referred to as diagonal, propeller, or lateral (Fig. 1E) [13, 14]. The presence of bulges within strands between different layers of the G-quartets adds another level of complexity and is considered as another target for drug discovery [15, 16]. Throughout this review, double-strand DNA will be abbreviated as ‘dsDNA’, a G-quadruplex as ‘G4’, and a ligand/G-quadruplex complex as ‘ligand/G4’ (where the term ‘ligand’ can be replaced by the specific name for the complex).

## The roles of G-quadruplexes in biology and cancer

Not only are G-quadruplexes often found in human telomeric ends of genes [6], they are also particularly abundant in the promoter regions of oncogenes such as *c-Myc*, VEGF, *c-Kit*, and *Bcl-2* (sequences shown in Fig. 1B) [17]. Importantly, 85% of all forms of cancer telomerases are overexpressed [18], which results in poorly controlled elongation of telomeric regions and increases the lifecycle of a cell. This poorly controlled cell division can be inhibited when the telomeric ends of genes and/or promoter regions of oncogenes fold into stable G-quadruplexes that cannot be copied [14]. Therefore, G-quadruplexes are appealing targets for therapeutic agents. In addition, *c-Myc* in particular can induce the expression of telomerase reverse transcriptase (TERT) mRNA, which leads to increased telomerase activity, and suppressed expression of this oncogene shortens the lifecycle of a cell [19].

## Metal complexes as G-quadruplex-stabilizing ligands

In view of the various roles of G-quadruplexes in biology and the occurrence of cancer, small compounds with high affinity and specificity for G4-DNA are of interest as novel therapeutic agents, in particular cancer [20]. Such species could stabilize, or even induce, the formation of G4s, thereby preventing enzymatic elongation of DNA or the expression of oncogenic proteins [20]. Although many small organic molecules have been identified as G4 binders, metal complexes offer features that are not available to organic molecules [8]. First, metal complexes have distinct structural and chemical features such as redox activity, reversible coordinating bonds, and the presence of atomic interactions that are more flexible than strict covalent bonds [8]. In addition, they offer a wider variety of subtly different geometries due to the coordination geometry around the metal center (e.g., planar, tetrahedral, pyramidal and octahedral) than present in most organic molecules. This variation in geometries results in a larger array of potential binding modes [8]. Second, a library of metal complexes can often be obtained by minor changes in the synthetic schemes, resulting in metal complexes that display finely tuned properties that translate into different biological effects. This is nicely illustrated by metal–salphen complexes of Karim et al. [21] (vide infra). A third advantage of metal complexes is the cationic nature of the metal–ion. In general, this leads not only to enhanced membrane permeability, which is advantageous for DNA-binding drugs that have to penetrate both the plasma and the nuclear membrane, it also enhances electrostatic interactions between the metal complex and the negatively charged phosphate backbone of the G4 [8]. A fourth and last advantage, which is only harvested by some metal complexes, are intrinsic features that can be utilized for activity regulation and analysis, such as magnetic properties, unique atomic composition, and unique spectroscopic features [22] or fluorescence profile to identify and visualize the compound inside a cell [23].

## Methods to study G-quadruplex stabilizers

Accurate determination of the ability of metal complexes to stabilize a G4 is crucial. In this section, we address several methods that have been used, including in vitro and in vivo bioactivity studies. Here, we emphasize that the aim of this review was to correlate the results of the various analytical techniques (Tables 1-5, and Table 6 for an overview) to the biological data obtained by in vitro (and potentially in vivo) studies (Tables 7 and 8), in order to provide guidelines or maybe even a framework for future research on this important topic. Complexes that meet these criteria are provided in Tables 7 and 8. For more comprehensive reviews on the application of metal complexes as potential G4-binding ligands, the reader is referred to more exhaustive reviews [8, 9]. As such, we briefly treat the various techniques used to analyze the interactions between metal complexes and G4, and mention advantages and limitations. It will become clear that a golden analysis technique does not exist, and that a combination of methods in combination with appropriate biologically relevant experimental conditions is needed for a better understanding and prediction of the G4 interactions that one aims for in relevant biological systems.

A general aim for most studies is the determination of the affinity of a ligand for a particular G4 forming sequence. This can efficiently be determined by measuring the melting temperature of the ligand/G4 complex, and comparing the result with that of the isolated G4. The melting temperature *T*_m_ is defined as the temperature at which 50% of the G4 structure is unfolded. As a higher *T*_m_ indicates a more stable G4, a higher *T*_m_ in the presence of a ligand indicates that it stabilizes the G4 [24]. The ability to stabilize the G4 is reported by ∆*T*_m_, which is calculated by subtracting the *T*_m_ associated with the isolated G4 from that of the ligand/G4 complex. To assess whether the G4 is melting, spectroscopic analysis such as UV–vis absorption, circular dichroism (CD), or Förster resonance energy transfer melting (FRET-melting) proved most useful. As *T*_m_ is also affected by solvent composition, pH, concentration of both ligand and G4, it is rather difficult to compare results from different studies from the different labs. To counter this and to obtain melting temperatures that better reflect conditions in biological systems, Li and his group investigated whether it is possible to induce G4 structures in vitro under conditions similar to those found in vivo [25]. They observed that G4 structures were formed from duplex DNA at near physiological salt concentration (150 mM K^+^), temperature (37 °C), and pH (7.4) in the presence of molecular crowding agent PEG (400 g/L) [25]. Such conditions offer an opportunity to reveal the potential biological benefit of these drugs more accurately than when purified solvent systems are used in the studies [25]. Nevertheless, the ∆*T*_m_ is a convenient and rapid indication to compare various G4s within one study, assuming the relevant experimental conditions are identical [30].

### High-resolution structural methods

#### Nuclear magnetic resonance (NMR) spectroscopy

This technique has proven useful for studying the structural, dynamics and kinetics of G-quadruplexes [26] and of ligand/G4 complexes on a broad time scale (picosecond–seconds) [27] (Table 1). Whereas detailed structural information has been obtained on isolated G4 structures and some of their complexes in purified solvent systems, more recently ligand/G4 complexes have been studied under in vitro conditions, providing not only insight in the effects of the environment on the ligand/G4 complex formation, but also revealing off-target interactions with genomic DNA [28]. More recently, studies were performed using conditions that are closer to living cells while maintaining the required atomic resolution [29]. An important limitation of this technique, however, is that it requires substantial amounts of material and that it provides limited insights in the driving forces for a given interaction [26].

#### Single-crystal X-ray crystallography

This technique provides detailed spatial and structural characterization of ligand/G4 interactions, even down to the atomic level [30] (Table 1). Although electrostatic interactions and hydrogen bonds can be quantified and visualized, this technique does not provide insight in the driving forces for complex formation as only the endpoint is visualized. In addition, substantial amounts of material are needed, the complex needs to crystallize under biologically relevant conditions, and it remains to be determined which elements of the provided structural insights are relevant for the biological activities. Nevertheless, the elevated level of details could enable the design of more potent ligands, which is hardly possible by other methods.

### Low-resolution structural methods

#### Circular dichroism (CD)

This is often a pioneering method for the evaluation of the binding properties of metal complexes, and their effect on the folding and secondary structures of biomolecules [31, 32]. (Table 1) It is preferred during the initial stages of ligand/G4 characterization as it is fast, requires small amounts of sample, and is relatively simple and inexpensive [31]. It provides preliminary information on the ligand/G4 complex and titrating the ligand to the G4 solution provides insight in the changes that occur in the conformation of the G4 in the presence of a ligand [32]. Once a set of ligands has been identified for further study, more detailed information can be obtained from NMR titration experiments and two-dimensional analysis of the various stages (Tables 1, 2, 3, 4, 5 and 6).

**Table 1:** Overview of the various methods for the evaluation of the affinity of metal complexes for G-quadruplex DNA on a structural level

Method	Advantages	Limitations
X-ray crystallography	Atomic resolution of the complex	Expensive equipment Large amount of sample Crystallizable ligand/G4 required Sensitive to differences in conditions
NMR	Broad timescale Variable temperature Atomic resolution of the complex Sensitive to subtle changes Fast	Expensive equipment Large amount of sample
CD	Very fast Easy to use Small amount of sample Sample not damaged Inexpensive	Additional methods needed for full characterization Interpretation is sometimes ambiguous

**Table 2:** Overview of the various methods for the evaluation of the affinity of metal complexes for G-quadruplex DNA on a biophysical level

Method	Advantages	Limitations
UV–vis absorption	Accurate Easy to use Inexpensive equipment Fast measurements	Additional methods needed for full characterization Interpretation is sometimes ambiguous
FRET-melting	Fast Easy to use Inexpensive equipment Small amount of sample Real-time monitoring	Not suitable for unstable G4s Fluorescently labeled oligonucleotides needed
ITC	Detailed thermodynamic information about the interaction Directly measures the affinity	Expensive equipment Large amount of sample
SPR	Valuable information interactions Directly measures the affinity	Expensive equipment Hard to precisely control Heterogeneous system Chemical modification required
MS	Valuable information interactions Directly measures the affinity	Expensive equipment Hard to precisely control Potentially changes G4 structures in vacuum
Equilibrium dialysis	Easy to perform Inexpensive Sensitive at low-affinity levels	Long timeframe for experiment Complex breakdown within timeframe

**Table 3:** Overview of the various methods for the evaluation of the affinity of metal complexes for G-quadruplex DNA on a physical level

Method	Advantages	Limitations
Affinity chromatography	Fast and convenient Real-time monitoring Promising for in vivo purposes	Immobilization of ligand or G4s required No structural information
G4-FID assay	Does not require modified oligonucleotides Fast and convenient Small amount of sample	Probes can be unspecific and unselective
Microarray	Fast and convenient Small amount of sample High throughput	Fluorescently labeled molecules Expensive equipment and analysis Immobilization of ligands and G4s

**Table 4:** Overview of the various methods for the evaluation of the affinity of metal complexes for G-quadruplex DNA on an enzymatic level

Method	Advantages	Limitations
qPCR-stop assay	Fast High throughput Fits the desired biological function of the complex	Expensive equipment Complex to perform
TRAP assay	Sensitive High throughput Fits the desired biological function of the complex Indicator for in vivo efficiencies	Not always accurate Complex to perform Expensive equipment

**Table 5:** Overview of the various methods for the evaluation of the affinity of metal complexes for G-quadruplex DNA on a biological level

Method	Advantages	Limitations
MTT assay (in vitro)	Relatively inexpensive Analysis in a few days Golden standard for cytotoxicity	Limited sensitivity MTT can be toxic to eukaryote cells Known chemical interferences
In vivo biotoxicity	Determine potency of drug Evaluate safety, toxicity and efficacy	Expensive Approval ethical board required Discrepancy results animals for humans Long timeframe

**Table 6:** Overview of the various methods for the evaluation of the affinity of metal complexes for G-quadruplex DNA

Method	Advantages											
	Speed	Convenience	Price	Accuracy	Sample amount	Reliability	Molecular details	Sensitivity	Thermodynamic details	Relevance for biology	Level of control	Intact sample
X-ray crystallography				X		X	X					
NMR	X	X		X		X	X	X			X	
CD	X	X	X		X							X
UV–Vis absorption	X	X	X	X								
FRET-melting	X	X	X		X						X	
ITC				X		X			X			
SPR						X			X			
MS						X			X			
Equilibrium dialysis		X	X					X				
Affinity chromatography	X	X		X						X		
G4-FID assay	X	X			X							X
Microarray	X	X			X							
qPCR-stop assay	X									X		
TRAP assay		X						X		X		
In vitro MTT assay	X		X							X		
In vivo biotoxicity				X		X				X		

### Affinity-based methods

Direct determination of the affinity of a metal complex for the G4 is preferred, especially if it can be compared with affinity studies using other types of DNA. In many cases, affinity constants (*K*_a_) can be determined using isothermal titration calorimetry (ITC), equilibrium dialysis (ED), mass spectrometry (MS), and surface plasmon resonance (SPR) (Table 2). Of these, ITC, ED and MS do not require any chemical modification on either ligand or G4, whereas SPR requires potentially disturbing immobilization of one of the two binding partners on a surface [33]. When sufficient material is available, ITC is preferred as it provides the full thermodynamic profile of the interaction [34]. In case less material is present, MS can be used although switching from a biosimilar buffer system to a MS-compatible buffer can induce changes in the G4 structure and thereby affect binding of the ligand [35]. Whereas SPR, ITC and MS are expensive methods, ED is inexpensive. Another advantage of ED is its sensitivity at even low-affinity levels, although it requires long incubation times of roughly twelve hours in order to allow for a proper thermodynamic equilibrium [36].

### Affinity chromatography

In contrast to MS, SPR and ITC, affinity chromatography is relatively fast and a simple method to determine the affinity of a ligand for a G4 (Table 3). Affinity chromatography requires immobilization of the G4 on a surface in a fashion that enables it to bind to ligand that is eluted over the column [37, 38]. In principle, the method is compatible with a biomimetic environment and the reverse process (in which ligands are immobilized) can be applied to bind G4s present in the eluent formed by complex mixtures and even derived from biological samples [39].

### Fluorescent intercalator displacement

For the affinity of a G4-binding complex using a Fluorescent Intercalator Displacement (G4-FID) assay, the loss in fluorescence upon displacement of thiazole orange (TO) from the G4 by the newly designed G4-ligand is measured [40] (Table 3). The concentration of ligand needed to displace 50% of the thiazole orange is reported as the DC_50_-value. However, as TO also binds to dsDNA, the concentration at which 50% of TO is displaced from dsDNA needs to be determined as well, and the ^G4^DC_50_ and ^dsDNA^DC_50_ values need to be compared [39]. Whereas the ^G4^DC_50_ or ^dsDNA^DC_50_ value gives an indication of the affinity of a metal complex for G4 or dsDNA, respectively, the ^dsDNA^DC_50_/^G4^DC_50_ ratio is a measure for the selectivity of the complex for G4s over dsDNA (< 1 indicates G4 selectivity) [40] (see Table 7).

**Table 7:** Physical properties of G-quadruplex-stabilizing metal complexes mentioned in this review

Complex	^*hTel*^IC_50_-TRAP (μM)	^G4^IC_50_-PCRs (μM) {^dsDNA^IC_50_/^G4^IC_50_}	^G4^DC_50_ (μM){^dsDNA^DC_50_/^G4^DC_50_}	Selectivity (K_G4_/K_dsDNA_)
**1-Ni**^**2+**^	5	–	–	2.0∙10^–1^ (*hTel*)
**1-Mn**^**3+**^	25.9	–	–	2.4∙10^1^ (*hTel*)
**2**	0.58	–	–	1.0∙10^4^ (*hTel*)
**3**	0.25	–	–	–
**5-Zn**^**2+**^	0.02	–	–	–
**6**	–	–	–	5.0∙10^3^ (*hTel*)
**9-Cu**^**2+**^	–	3.51 {2.02} (*hTel*) 2.74 {1.53} (*c-Myc*)	–	6.4∙10^1^ (*hTel*)
**9-Mn**^**3+**^	–	2.37 {2.72} (*hTel*) 1.52 {2.13} (*c-Myc*)		4.9∙10^1^ (*hTel*)
**10**	0.14	–	0.20 {–} (*hTel*)	1.4∙10^3^ (*hTel*)
**11-Ni**^**2+**^	–	19.2 {–} (*hTel*)	0.33 {–} (*hTel*)	4.6∙10^2^ (*hTel*)
**11-Cu**^**2+**^	3.6	–	0.36 {–} (*hTel*)	5.9∙10^1^ (*hTel*)
**12**	4.8	–	0.26 {–} (*hTel*)	1.4∙10^2^ (*hTel*)
**13**	–	4.4 {–} (*hTel*)	–	9.0∙10^0^ (*hTel*)
**14**	–	–	–	–
**15**	–	0.2 (Pu22myc)		4.9∙10^1^ (*hTel*) 3.5∙10^1^ (*c-Myc*)
**16**	–	–	–	3.2∙10^2^ (*hTel*)
**17**	0.76	–	–	8.1∙10^2^ (G4A1)
**18**	–	–	–	2.7∙10^2^ (*hTel*)
**19**	–	–	–	1.6∙10^2^ (*hTel*)
**20**	–	–	–	6.0∙10^2^ (*hTel*)
**21**	–	–	0.3 (22) (*hTel*)	–
**22**				1.6∙10^2^ (*hTel)*
**23**	3.0	–	–	–
**24**	–	–	0.01 {150} (*hTel*)	–
**25**	1.9	–	0.5 {> 10} (*hTel*)	*n.d.*^b^
**26**^a^	0.24	–	0.52 {186} (*hTel*)	–
**27**	0.3	0.15 {–} (HTG21)	–	3.6∙10^1^ (*hTel*) 1.1∙10^0^ (bcl2)
**28**	0.12	1.5 {–} (HTG21)	–	1.19∙10^2^ (*hTel*) 3.8∙10^0^ (*c-Myc*) 1.1∙10^1^ (bcl2)
**29**	–	–	0.08 {19} (*c-Myc*) 0.35 {4.3} (*hTel*)	7.2∙10^2^ (*c-Myc*) 4.8∙10^1^ (*hTel*)
**30**	5	–	0.91 {–} (Pu27^d^)	–
**31**^a^	0.9	–	0.55 {59} (Pu27^d^) 2.24 {13} (c-kit-1) 3.46 {9} (c-kit-2) 1.31 {25} (HTG21) 1.52 {21} (Pu39^e^) 1.94 {17} (Pu22^d^)	–
**32**^a^	–	–	0.89 {110} (Pu27) 2.52 {39} (c-kit-1) 1.22 {80} (HTG21)	–
**33**		< 2 μM		

Selectivity of the metal complex to bind to dsDNA (> 1) or G4 (< 1) is given by the numbers between the accolades {}. The code of the gene from which the G4 sequence was derived is given between the brackets ()

^a^These compounds have also been tested in vivo

^b^Binding constant with dsDNA too low to measure

^c^The desired parameter could not be obtained from these results

^d^part of the c-Myc sequence

^e^part of the Bcl-2 sequence, regulates cell death by apoptosis

### Microarray-based methods

Microarrays are used for high-throughput purposes in which thousands of immobilized G4-DNA sequences can be screened for one particular metal complex, especially in early-stage drug discovery [26, 41]. The reverse strategy is also possible where a library of immobilized ligands is screened against various G4 forming sequences [42]. These microarrays have some limitations accompanied as they rely on fluorescently labeled molecules, specialized equipment, immobilization of ligands and G-quadruplexes, and are expensive [26]. Moreover, microarray-based methods are only recently applied for characterization of G-quadruplex/ligand interactions and are still being optimized to exploit their full potential [26, 43].

### PCR-based methods

Whereas the aforementioned methods tolerate a limited biological influence, a method that taps into the desired ultimate biological function of a G4-stabilizing ligand is based on a polymer chain reaction (PCR) (Table 4). Specifically, the quantitative PCR (qPCR) stop assay and the Telomere Repeat Amplification Protocol (TRAP) assay are most commonly used. In the first assay, a G4-binding ligand is added to the DNA sequence after which the DNA is subjected to PCR chain lengthening. When the ligand is bound to the G4, the PCR is inhibited [44], and the higher the affinity of the ligand the lower the amount of G4-ligand that is needed to inhibit the amplification reaction. The ligand concentration at which the amplification is reduced by 50% is called the IC_50_-PCR value. Similarly, the TRAP assay relates to inhibition of telomerase, which elongates human telomeric DNA (*hTel*) and is, therefore, more relevant for anticancer research [45]. Here, the concentration of ligand required to reduce the telomerase activity by 50% is called the IC_50_-TRAP value. As a measure for the selectivity of the metal complex, the ratio ^dsDNA^IC_50_/^G4^IC_50_ for PCR or TRAP is used.

Next to the assessment of the interaction of metal complexes and distinct types of DNA in clean systems, their applicability as anticancer drug is better analyzed using biological studies such as in vitro cell viability studies and even in vivo tumor growth inhibition studies.

### In vitro* cytotoxicity*

Using cell lines obtained from human cancer tissues or from healthy tissues, toxicity of compounds when exposed to these cells can be determined. An MTT assay is a widely accepted method as indicator of cell cytotoxicity, proliferation and viability and can be considered as the golden standard for cell viability [46] (Table 5). The assay is based on metabolically active cells that can induce the reduction of a yellow tetrazolium salt (MTT) into purple formazan crystals [47]. Although MTT assays are often used, various chemical compounds—e.g., vitamin A, coenzyme A, and sulfhydryl-containing compounds—are known to affect enzymatic or chemical reduction of MTT, leading to higher background absorbance or to false positives [48]. To solve this, luminescent (e.g., luminescent ATP) and fluorescent (e.g., RealTime-Glo™) methods are generally more sensitive and offer a potentially better alternative [48]. In general, the concentration that reduces cell survival by 50% is reported as the IC_50_ value and by measuring the IC_50_ value using both cancer cells and healthy cells, an indication of the selectivity of the ligand/G4 complex is obtained. Unfortunately, as incubation time, cell type, number of passages that the cells underwent prior to the study, and many other variables influence the IC_50_ value, caution is warranted when comparing IC_50_ values from different studies. However, when a standard such as cisplatin is included in the study, comparison between different studies is more reliable. As a case in point, of the 74 metal complexes that we analyzed for this review, approximately 72 complexes have been subjected to in vitro analysis of their cytotoxicity [49], but only approximately half of these were compared to cisplatin in the same experiment.

### In vivo* biotoxicity*

To assess the therapeutic potential of the metal complex, in vivo studies are applied (Table 5). Especially biodistribution of the complexes reveal the extent to which the metal complexes end up in the targeted tissue. Unfortunately, only 3 of the 74 metal complexes have been tested in vivo [49], and the reported studies were performed on mice with or without artificial tumors for tumor growth inhibition and acute toxicity [50]. As alternative to mouse studies, biotoxicity studies could be performed on developing zebrafish embryos [51]. Whereas the mouse model provides insight in potential clinical applications, the zebrafish model enables rapid evaluation of toxicity of potential drugs due to short growth period, high fertility, and high reproductive rate.

## G-quadruplex-stabilizing metal complexes

**Fig. 2:**
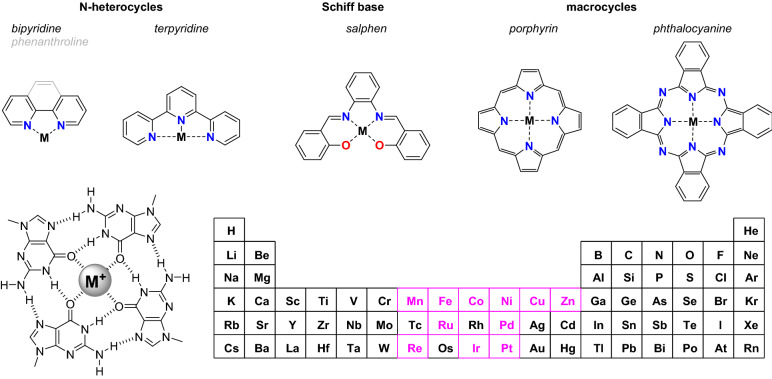
Overview of the metal complexes, including metals and ligand structures described in this review. The guanine tetrad that is the target of the ligands is shown in the same proportions as the ligands. The periodic table highlights the transition metals that have occurred in the metal complexes treated in detail in this review

To date, a fraction of G4-stabilizing ligands is metal based. Although many of these complexes can be found in the recently updated G-quadruplex ligand database (G4LDB 2.2) [49], we also included various complexes that were not listed there. Nevertheless, the G4LDB 2.2 provides some insight in the role of metal complexes as G4-stabilizing ligands as of the approximately 1400 G4-stabilizing ligands that were reported until 2021, 74 were metal-containing ligands (Fig. 2) [49].

### Metalloporphyrins

**Fig. 3:**
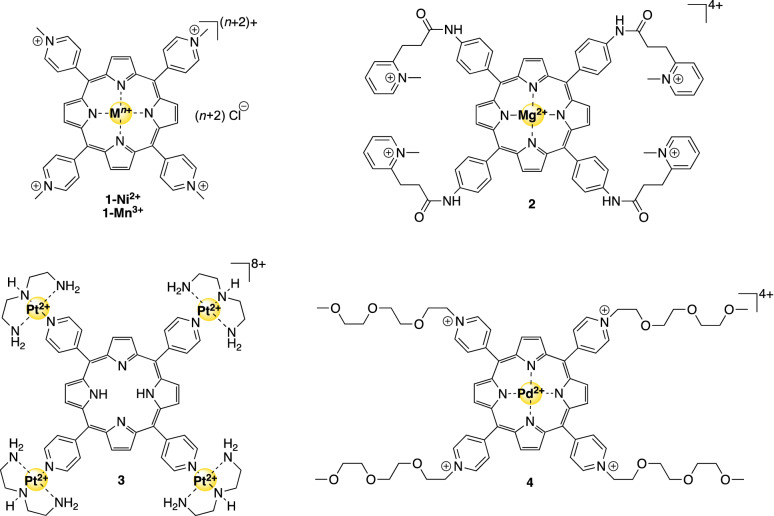
Structures of G4-binding metalloporphyrins mentioned in this review

Metalloporphyrins were among the first reported G4-stabilizing metal complexes, by Hurley and Meunier [52, 53]. At the moment, these are the most-studied inorganic G4 stabilizers [54]. These planar metal complexes preferably bind via π–π stacking onto the G-quadruplexes, making optimal use of their geometry, symmetry and size [55]. As the properties of porphyrins can be altered by changing the metal center or by modifying the *meso* substituents at the periphery of the porphyrin core, they have also been subjected to various optimization strategies. For example, the TMPyP4 porphyrin that contains a Ni(II) center (**1-Ni**^**2+**^, Fig. 3) already displayed an IC_50_-TRAP value of 5 µM [56]. Even though it preferred binding to dsDNA (selectivity of 0.2 for G4_*hTel*_), its preference for the G4_*hTel*_ structure could be increased by replacing the Ni(II) for the Mn(III) ion (as in compound **1-Mn**^**3+**^, Fig. 3), resulting in a 24-fold increase in binding selectivity in favor of G4_*hTel*_. It was suggested that the presence of the two axially coordinating ligands on the Mn(III) complex hampered dsDNA binding but favored binding to the G4_*hTel*_ structure [56].

A higher binding selectivity for G4 was obtained by a metalloporphyrin containing four flexible cationic arms, compound **2** (Fig. 3). As the bulky substituents prevented close interactions of the central core with dsDNA, favorable binding to the top of a G-quartet was obtained and a remarkably high binding selectivity of 10^4^ was determined [57]. In fact, it was proposed that the cationic arms simultaneously interact with the grooves and the loops of the G4_*hTel*_ structure, resulting in even higher affinities than could be expected based on the metalloporphyrin core alone [57]. As expected, this compound showed promising results in the TRAP assay, with an ^*hTel*^IC_50_-TRAP value of 0.58 µM, beaten only by a metal-free porphyrin-bridged tetranuclear Pt(II) clover (complex **3**, Fig. 3), which displayed an ^*hTel*^IC_50_-TRAP value of 0.25 µM [58]. Further analysis revealed that compound **3** is toxic towards various cancer cell lines in the micromolar range, and that it operates via oncogene repression and inhibition of telomerase activity [58]. Unfortunately, even though compounds **2** and **3** showed promising results in various in vitro assays, in vivo studies were not performed on these complexes. The fluorescent Pd-TEGPy metalloporphyrin **4** (Fig. 3) was explored as a potentially trackable drug in cells, although it was not explored in that setting [59]. This inherent feature of metal complexes avoids otherwise structural modifications to obtain fluorescent ligands, which could negatively impact G4 specificity. Future research is required in order to determine if these complexes are potential drug candidates or not.

### Metallophthalocyanines and metallocorroles

**Fig. 4:**
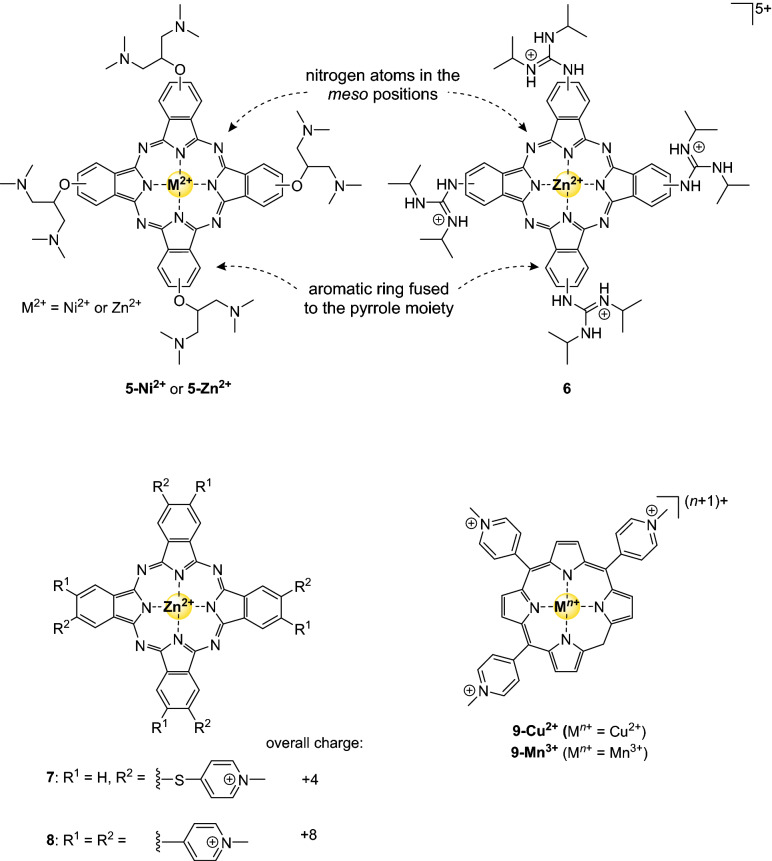
Structures of G4-binding metallophthalocyanins and metallocorroles mentioned in this review

The metalloporphyrin derivatives metallophthalocyanines and metallocorroles have also been explored as G4-stabilizing ligands. On the one hand, phthalocyanine is a porphyrin that has aromatic rings fused to the pyrrole moiety and nitrogen atoms in the *meso* position (Fig. 4), providing a larger aromatic surface that could favor π-stacking on top of a G-quartet. Corroles, on the other hand, resemble classic porphyrins, but have only three *meso* substituents and are typically saddle-shaped instead of the planar shape of metalloporphyrins. Zn(II) and Ni(II)-phthalocyanines (**5-Ni**^**2+**^ and **5-Zn**^**2+**^, respectively, see Fig. 4) with excellent IC_50_-TRAP values have been reported [60, 61]. So far, the strongest binding complex (**5-Zn**^**2+**^) displayed an ^*hTel*^IC_50_-TRAP value of 20 nM [60], which was attributed to a combination of the core-metal, the substituents, and the number of positive charges. Although the ability to inhibit telomerase was investigated, their selectivity towards G-quadruplex DNA and their cytotoxicity remained undetermined. In general, nickel phthalocyanines were more active than zinc phthalocyanines, which was attributed to differences in electrostatic and subtle structural factors [60, 62]. Specifically, nickel complexes are more planar than the corresponding zinc complexes, which likely results in a more optimal interaction between the delocalized π-electrons of the ligand and of the G4 [62].

Metallophthalocyanine Zn-DIGP complex **6** preferred *c-Myc* quadruplexes over duplex DNA with a selectivity factor of 5000 and was able to knockdown RNA-expression of *c-Myc* in neuroblastoma cells [63]. In addition, it was used as probe for *c-Myc* G4 imaging as binding to the G-quadruplex increases the luminescence of the complex substantially.

As the number of positive charges on substituents affected the ability of compounds to inhibit telomerase, it was suggested that compounds with an increasing number of positive charges should also result in a higher activity due to the additional electrostatic interactions with the negatively charged DNA [60]. Recently, phthalocyanines that bear four or eight positive charges confirmed the importance of charge for the selectivity and affinity towards the G-quadruplexes [64]. Compounds ZnPc1 (complex **7**) and ZnPc4 (complex **8**) showed high affinity and selectivity, and an ability to accumulate in desired cancer cells.

The best G4-binding metallocorroles reported so far were compounds **9-Cu**^**2+**^ and **9-Mn**^**3+**^ (Fig. 4). The IC_50_-PCRs values of these two compounds were in the single-digit micromolar range with 3.5 µM and 2.4 µM for *hTel* and 2.7 µM and 1.5 µM for *c-Myc*, respectively. Moreover, moderate selectivities (^dsDNA^IC_50_/^G4^IC_50_) of 2.0 and 2.7 for *hTel* and 1.5 and 2.1 for *c-Myc* were obtained, respectively, as well as a binding selectivity for G4_*hTel*_ over dsDNA of 49 and 64, respectively [65]. As these metallocorroles are characterized by effective stabilization of transition metal ions in high oxidation states, the formation of saddle-shaped geometries probably limits the selectivity for G4 structures that can be gained [65, 66].

### Metal–salphen complexes

**Fig. 5:**
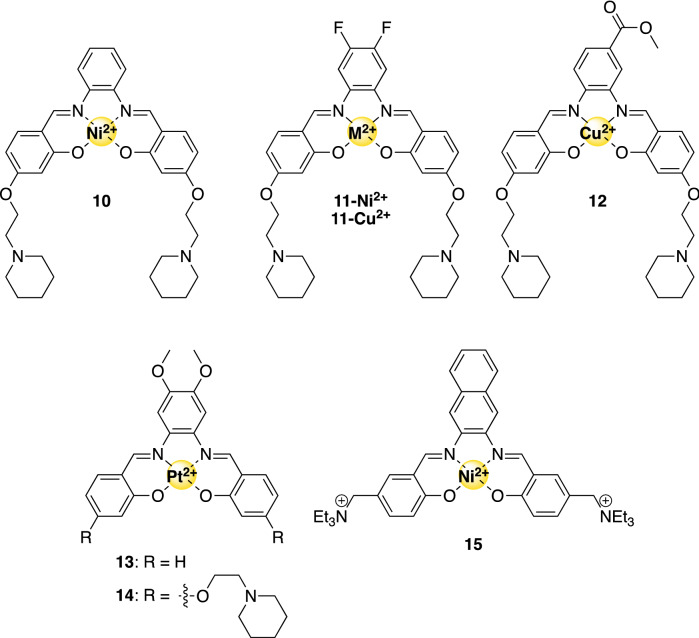
Structures of G4-binding metallo-salphen complexes mentioned in this review

Metal–salphen complexes are relatively small organic compounds that consist of a symmetrical metal-binding ligand that includes two phenol groups and two imines as metal-binding moieties (see Figs. 2, 5). The pioneering work of Vilar et al. showed that metal–salphen complexes have a remarkable ability to stabilize G-quadruplex DNA structures [62, 67]. As a result, metal–salphen complexes containing a broad range of metal ions have been evaluated with respect to their binding affinity towards G4 and their toxicity to various cells [68–70]. The complexes exhibit excellent selectivity towards the G-quadruplexes over the dsDNA, which was mainly through the addition of positively charged groups or by introducing specific DNA recognition moieties [71]. In addition, insertion of different metal ions in the salphen ligand led to complexes with slight differences in the planar shape, even with hints towards a pyramidal geometry in the presence of an additional coordinating ligand (vide infra) [21]. The resulting complexes showed remarkable differences in biological activities that could be correlated to the geometry of the metal complex.

Metal–salphen complexes **10** and **11-Ni**^**2+**^ bind to G-quadruplexes and the single crystal X-ray structure of the Ni(II)-salphen complex **10** bound to ^*hTel*^DNA revealed that it binds by end-stacking with the Ni(II) ion positioned on the central axis of the G4 [68]. Both complexes displayed very good activities in G4-FID, PCR, and binding studies [62, 68]. Unfortunately, these compounds were not only toxic against cancer cells (IC_50_ = 2.3–3.3 µM), but they also displayed poor in vitro selectivity (relevant cell lines are mentioned between brackets): 1.0 (HepG2) and 1.16 (HeLa) for **10** and 2.5 (MCF7) and 7.5 (A549) for **11-Ni**^**2+**^ (see Fig. 5 for structures).

Two similar complexes **11-Cu**^**2+**^ and **12** (Fig. 5) displayed a lower affinity towards *hTel* G4-DNA than complexes **10** and **11–Ni**^**2+**^ [68]. Crystallographic analysis of the complexes themselves showed that the bending of the Cu(II)-complex is larger than that of the Ni(II)-complex, resulting in suboptimal interaction with the G-tetrad plane and the lower affinity. Nevertheless, in vitro selectivity of the Cu(II)-salphens is better than that of the Ni(II)-salphen complexes, with values of 2.6 (determined in MCF7 cells) and 4.9 (determined in MCF7 and HeLa cells), respectively. Although it was suggested that complexes **11-Cu**^**2+**^ and **12** stabilize G-quadruplexes that are situated in a promoter region of an oncogene (e.g., in *c-Myc*), it could also be that uptake of these compounds by the cells was more efficient than for the corresponding Ni(II) complexes.

Two Pt(II)-based G4-ligands **13** and **14** (Fig. 5) proved potent anticancer therapeutics based on their cytotoxicity cell tests [70]. Compound **13** was more selective towards cancer cells with a factor 7.0 (against HeLa cells) and 10.5 (against HepG2 cells), whereas cisplatin was much less selective against the same cell lines with selectivity values of 1.0 and 1.4, respectively. Since it was observed that compound **13** stabilized *c-Myc* G-quadruplexes better than compound **14**, resulting in more potent inhibition of *c-Myc*-expression, it was suggested that these compounds probably have a different target, and that the activity of **14** is not only caused by G4 stabilization.

In order to assess the effect of additional aromatic rings as substituents, three metal ions were investigated in a complex of type **15** [68] (Fig. 5). Of the complexes with Ni(II), Cu(II) and Zn(II), the one with nickel was the most efficient. Compound **15** showed a high binding selectivity for G4-DNA *hTel* and *c-Myc* over dsDNA of approximately 49 and 35, respectively [69]. It was suggested that the extended aromatic ring system increased the stacking interaction with the G4 structures in *hTel* as well as in *c-Myc*. Moreover, the IC_50_-PCR value of this Ni(II) compound is 0.2 µM. Even though the cytotoxicity of compound **15** towards cancer cells was in the low micromolar range—i.e., 10–17 µM within 24 h and of 0.3–1.4 µM after 48 h—toxicity towards healthy cells was not tested, unfortunately.

### Planar metal–phenanthroline complexes

**Fig. 6:**
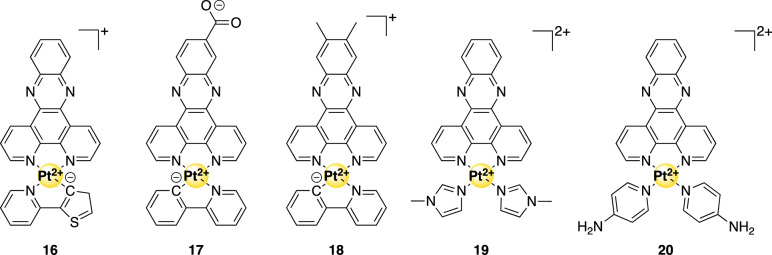
Structures of G4-binding metal–phenanthroline complexes mentioned in this review

The positively charged planar metal–phenanthroline complexes have a geometry for optimal binding to the flat ends of a G4 unit. Indeed, metal–phenanthroline complexes **16**–**20** (Fig. 6) appear to be selective binders for G4-DNA and selectively inhibit the growth of cancer cells [72]. Within this group, in vitro selectivity followed binding selectivity for *hTel* G4-DNA. It appeared that a more extended delocalized π-surface favors G4 interaction. The most promising metal–phenanthrolines were compounds **17** and **20**, with in vitro selectivity values of 10.3 and 8.1 (both against HeLa cells), respectively. Even more, the effectiveness of these complexes was 0.9 and 0.73 times less than that of cisplatin (also against HeLa cells), respectively. Additional evidence for compound **17** as an excellent inhibitor of *hTel* was obtained from an IC_50_-TRAP assay that revealed a value of 760 nM.

### Metal–terpyridine complexes

**Fig. 7:**
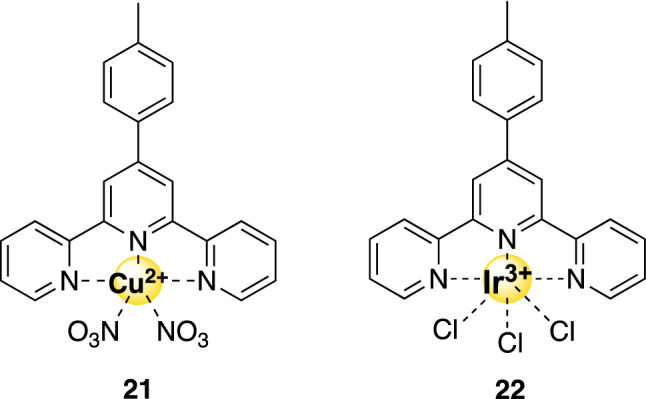
Structures of G4-binding metal–terpyridine complexes mentioned in this review

Teulade-Fichou et al*.* pointed out that the metal geometry is very important for recognition of the G-quadruplex DNA by metal–terpyridine complexes [73]. Metal–terpyridine complexes with the metal ions Zn(II), Cu(II), Pd(II), Pt(II), Ru(II), or Ir(II) can thus stabilize G-quadruplexes, despite the fact that they do not form the planar structures described above. In fact, it became clear that the geometry of the metal center strongly contributes to the selectivity for G4-DNA over dsDNA. Due to this deviation from the flat geometry, most complexes perform suboptimal [73]. For example, the Zn(II) complexes are not planar and are, therefore, less favorable for G4-binding. More promising complexes are compound **21** and **22** (Fig. 7). On the one hand, Cu(II)-terpyridine **21** adopts a pseudo-square-pyramidal structure and has a strong affinity and selectivity for G-quadruplex DNA according to FID. Although its in vitro selectivity has not yet been determent using healthy cells, it is a highly toxic compound for various cancer cell lines. On the other hand, compound **22** has been studied more broadly on its biological implications [74]. The Ir(II)-complex is able to downregulate expression of *c-Myc* and *hTERT*, important regulators of telomerase activity. Cytotoxicity cell tests on HepG2 cells showed that compound **22** had an in vitro selectivity of 9.6. Interestingly, the toxicity could be regulated by moving the methoxy-group: in the *para-* or *ortho-*position, the complex displayed lowered activity than the *meta*-position. These differences were attributed to differences in cellular uptake.

### Octahedral ruthenium complexes

**Fig. 8:**
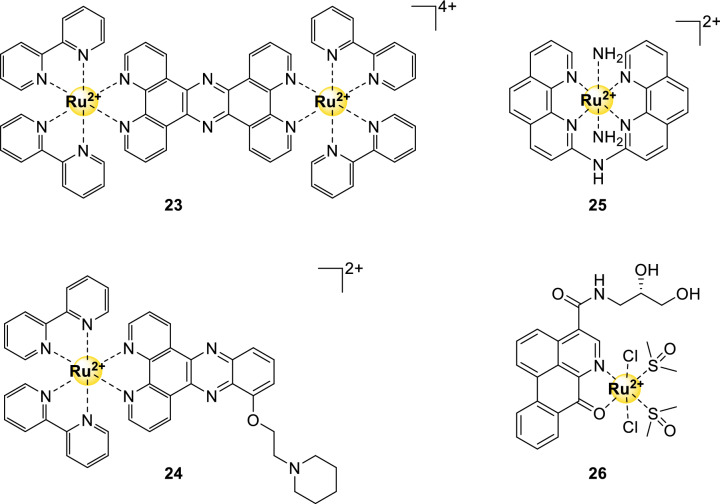
Structures of G4-binding octahedral ruthenium complexes mentioned in this review

Although simple octahedral ruthenium complexes are well known for their fluorescent properties, more intricately designed complexes displayed appealing G4-DNA-binding abilities. The pioneering work of Thomas et al*.* in 2006*,* on dinuclear monointercalating octahedral Ru(II)-complexes, revealed the potential of these complexes as it showed high binding affinity for G4-quadruplex DNA over duplex DNA [75]. Ru(II)-complex **23** (Fig. 8), in particular, had a binding selectivity for G_4*hTel*_ over dsDNA of 1600. This initial work on octahedral Ru(II)-complexes was further investigated in 2012, where they focused on the enantiopure isomers of these complexes instead of the diastereomeric mixtures [76].

From the reported octahedral Ru(II)-complex G4 stabilizers, a large percentage has been investigated on its cytotoxicity towards cancer cells [77]. Even though compound **24** displayed low toxicity immediately after administration, its toxicity increased over the time course of 6 days. From FID studies it became clear that the Ru(II)-complex binds effectively to *hTel* DNA, thereby inhibiting telomerase once the telomere sequence is too short [78].

Ru(II)-complex **25** (Fig. 8) binds strongly and selectively to G4-DNA in the basket configuration (Fig. 1D) [79]. The compound has a large planar aromatic surface for π-stacking and two axially coordinating ammonia molecules that can form hydrogen bonds with the guanine residues. In addition to a high binding selectivity to this type of G4-DNA, this complex also displayed good in vitro selectivity being 7.8 (against MCF7 cells), 16.7 (against HeLa cells) and 23.5 (against A459 cells) times more active against different cancer cell lines when compared to healthy cell lines.

One of the most promising complexes is compound **26** (Fig. 8), which could even compete with cisplatin in an in vivo model [77]. The strong affinity of this compound for G-quadruplexes was attributed to the nature of the chiral extension that was attached to the complex. The *S*-(–) isomer (structure **26**) displayed significantly higher activities than the *R*-(+) isomer, bound to *hTel* and *c-Myc* DNA, and resulted in cell senescence and apoptosis in an in vivo tumor mouse model. Although in vivo safety appears to be better than cisplatin, its activity to inhibit tumor growth is slightly lower than that of cisplatin.

### Multinuclear metal assemblies and dimetallic complexes

**Fig. 9:**
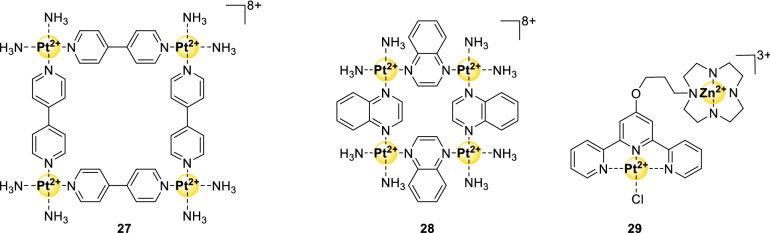
Structures of G4-binding multinuclear metal assemblies and dimetallic complexes mentioned in this review

Apart from the mononuclear complexes described above, multinuclear metal assemblies have been reported as G4 stabilizers as well. Although selectivity for targeting cancer cells is not known for these complexes, promising binding affinity and selectivity for G4-DNA sequences was shown [80, 81]. Specifically, multinuclear complexes **27** and **28** (Fig. 9) are square planar Pt(II) complexes that display excellent IC_50_-PCR values for *hTel* DNA of 0.15 µM and 1.5 µM, respectively. Similarly, they can inhibit telomerase excellently as indicated by the low ^*hTel*^IC_50_-TRAP value of 0.3 and 0.12 μM for **27** and **28**, respectively [58, 80, 81].

A more complicated multinuclear complex is the terpyridyl metal complex **29** [82] (Fig. 9). This complex interacts rather strongly and is selective for *c-Myc* DNA, but also has affinity for *hTel* DNA. The high binding selectivity of 720 for *c-Myc* is probably due to two types of interactions that are caused by this construct: the platinum–terpyridine complex can bind via end-stacking on the G-quartet, and the zinc-cycle part can interact with loops of the G4.

### Miscellaneous complexes

The dsDNA-binding cisplatin complex has inspired the development of platinum derivatives that bind specifically to G4-DNA. In general, these derivatives contain one or two labile groups allowing platinum to coordinate with the G4-DNA.

**Fig. 10:**
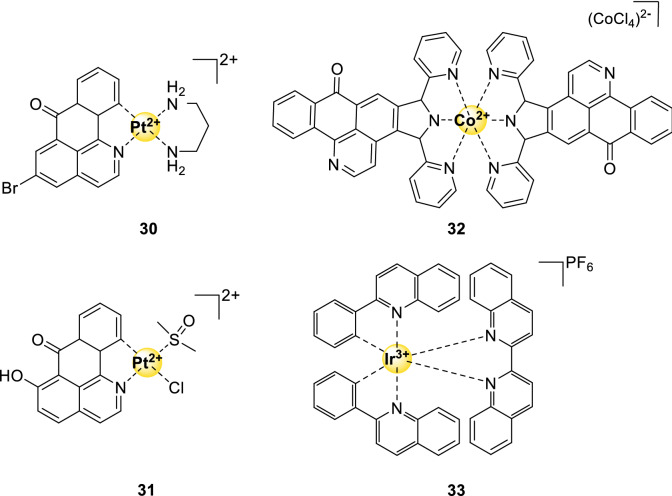
Structures of G4-binding miscellaneous complexes mentioned in this review

Two derivatives are promising anticancer therapeutics, both containing a dibenzoquinolinone ligand. Complex **30** (Fig. 10) targets various cancer cells 5.6 (against MCF7 cells), 15.4 (against HepG2 cells) and 37.4 (against A549 cells) times better than cisplatin itself [83]. It inhibits telomerase via targeting *c-Myc* DNA and also disrupts the mitochondrial dysfunction pathway, resulting in apoptosis. The antitumor activity of complex **31** (Fig. 10) has been studied in vitro as well as in vivo within a xenograft mouse model [84]. The in vitro selectivity of compound **31** is 4.2 (against HepG2 cells) times higher than that of cisplatin, which is probably associated with its strong binding to G4. On top of that, compound **31** exhibited high safety in mice and had a higher inhibitory effect on tumor growth than cisplatin. The toxic effect on cancer cells arises via inhibition of telomerase activity due to interaction with *c-Myc, hTel*, and *Bcl-2* G4-DNA [83].

Apart from the above-mentioned complexes [85–87], G4-stabilizing anticancer therapeutic agent complex **32** (Fig. 10) does not belong to any of the above groups [85]. It is one of the two cobalt-complexes reported as G4-ligand. FID assays showed that **32** binds strongly and specifically to various G4-DNA sequences (e.g., ^G4^DC_50_ = 0.89 µM and ^dsDNA^DC_50_/^G4^DC_50_ = 110 for Pu27, which is part of the *c-Myc* DNA sequence). It targets cancer cells in vitro (against HepG2 cells) 14 times more specific than cisplatin via downregulating the expression *c-Myc, hTERT*, and *Bcl-2* with 73%, 52%, and 90%, respectively, and its in vivo inhibition of tumor growth was as good as that of cisplatin.

In 2013, the first G-quadruplex stabilizer with an iridium(III) metal center (**33**, Fig. 10) was designed by Ma et al.[88]*.* This cyclometallated iridium(III)-complex, containing a 2,2-biquinoline ligand, showed a moderate to high binding affinity (8.3∙10^5^ M^−1^) towards parts of the *c-Myc* sequence without any significant interaction with duplex DNA [88]. The complex was also examined in vitro (IC_50_ < 2 μM) where it showed again no significant interaction with dsDNA [88]. Complex **33** also showed promising cytotoxicity in the sub-micromolar range (0.2 μM) towards cancer cells, however, toxicity towards healthy cells was not examined. Iridium–metal complexes are nowadays mainly used as G-quadruplex selective probes, including complex **33**, because of their unique luminescent property [89–91].

## Concluding remarks

**Fig. 11:**
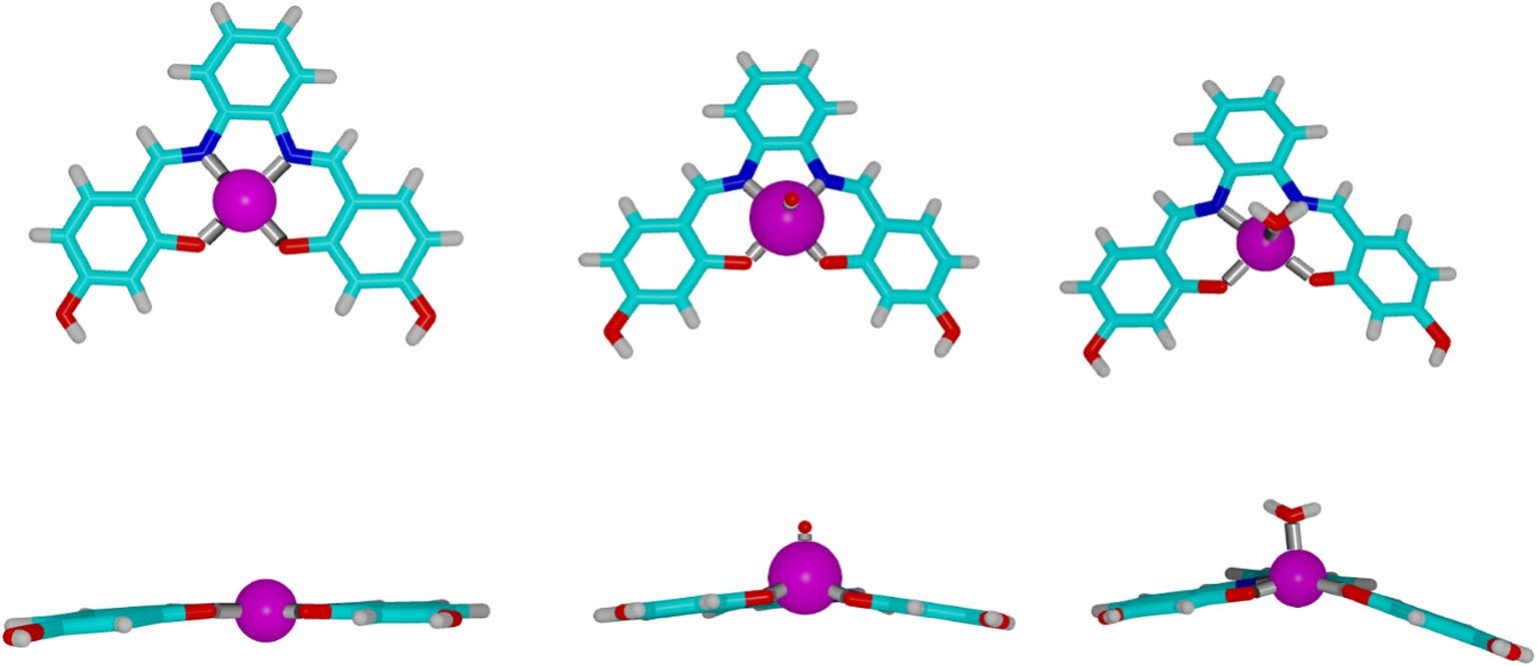
Metal–salphen complexes with Cu (left, CCDC-code 766686) [93], V (middle, CCDC-code 252952) [94], and Zn (right, CDCC code 667235) [95] in top and frontal view (top and bottom, respectively), with emphasis on the metal-dependent deviation from the flat nature of the complex. As the vanadium complex did not meet our criteria for this review, we did not highlight this element in the periodic table of the elements shown in Fig. 2

In this review, we analyzed G4-stabilizing metal complexes and extracted from this analysis guidelines for future studies. Herein, we focused on the comparison of outcomes for various techniques that have been used to characterize the interaction between ligands and G4s; for more comprehensive review of G4-stabilizing metal complexes we refer to the literature [92]. We noted that discrepancies between the techniques seriously complicated comparison of the activities of the different complexes. Experimental conditions were often not the same for various tested metal complexes (e.g., in melting temperature measurements), different promoter regions of oncogenes and parts of human telomeric DNA were used, and within in vitro cytotoxicity assays an array of different cancer cells were used. This will hamper progress for this important class of G4 stabilizers as it complicates assessment of the potential of particular metal complexes. Moreover, not only discrepancies in the techniques were observed, the absence of a golden analysis technique makes it inevitable to combine methods to obtain a better understanding of the G4-metal complex interactions. This is particularly unfortunate as metal complexes offer a level of fine-tuning for G4-binding that is hardly available using other types of ligands. For example, by simply changing one atom in the structure, i.e., the metal ion, the same organic framework can adopt different geometries (Fig. 11), each of which is associated with a different bioactivity. In view of these subtle differences between the different G4-binding ligands, it is not surprising that finding an appropriate balance between selectivity and G4 affinity appeared to be highly unpredictable. High affinities towards G4-DNA did not necessarily correlate to a high specificity towards cancer cells, and vice versa (Tables 7 and 8). This makes it even more crucial to extensively characterize G-quadruplex/ligand interactions as a restricted selection of techniques does not provide sufficient guidance.

**Table 8:** Ability of G-quadruplex-stabilizing metal complexes to selectively target G4s in cancer cells when compared to the effect of cisplatin in healthy cells; a higher number indicates a higher selectivity for the targeting of cancer cells

Complex	Cell line			
	HepG2	A549	HeLa	MCF7
**10**	1.0 (CD19LU, 48 h)		1.16 (CD19LU, 48 h)	
**11-Ni**^**2+**^		7.5 (WI38, 96 h)		2.5 (WI38, 96 h)
**11-Cu**^**2+**^		2.63 (WI38, 96 h)		
**12**		4.87 (WI38, 96 h)	4.87 (WI38, 96 h)	
**13**	10.54 {1.43} (CD19LU, 48 h)		7.03 {1.03} (CD19LU, 48 h)	
**14**	42.3 {6.0} (CD19LU, 48 h)		36.02 {5.4} (CD19LU, 48 h)	
**16**			7.79 {0.70} (CD19LU, 48 h)	
**17**			10.34 {0.93} (CD19LU, 48 h)	
**18**			6.67 {0.6} (CD19LU, 48 h)	
**19**			6.74 {1.64} (CD19LU, 48 h)	
**20**			8.1 {0.73} (CD19LU, 48 h)	
**23**	9.59 (HL7702, 48 h)			
**25**		23.53 (NIH-313)	16.67 (NIH-313)	7.84 (NIH-313)
**26**^a^	4.27 {2.6} (HL7702, 48 h)	6.41 {2.88} (HL7702, 48 h)	7.69 {2.86} (HL7702, 48 h)	
**30**	19.17 {15.41} (HL7702, 48 h)	11.44 {37.43} (HL7702, 48 h)		10.31 {5.57} (HL7702, 48 h)
**31**	6.94 {4.22} (HL7702, 48 h)			

The type of healthy cell line that is used in comparison as well as the reported incubation times are given between the normal brackets. Values between accolades {} represent toxicity of the respective metal complex in comparison with that of cisplatin against the cell line

^a^This compound has also been tested in vivo

A particular omission was the absence of biological data for G4-stabilizing metal complexes obtained in animal models. Despite their high potential as anticancer agents in cellular models of cancer (Table 7), it is of major importance to invest in the difficulties faced within clinical trials as well (e.g., drug delivery tools). As such, we were surprised that only a handful of complexes were also tested in vivo (Table 8). This suggests that this approach to cancer treatment is still rather infant, and that many promising complexes await to be discovered. In order to aid in this quest, we noted that the following characteristics led to potential anticancer drug: (i) Cationic arms that enable simultaneous interaction with the grooves and the loops of the G4 structures. (ii) Large aromatic surface to favor additional π-stacking on top of a G-quartet. (iii) Planar molecules display a more optimal interaction between the delocalized π-electrons of the ligand and of the G4, deviation from this flat geometry gave average results. (iv) DNA recognition moieties, potentially introduced by increasing specificity towards G4 structures. (v) Axially coordinated hydrogen bonding molecules that add hydrogen bonding opportunities with guanine residues. With this assessment, we hope that future research on metal complexes results in a more efficient and reliable exploration of the therapeutic potential of these interesting metal complexes.

## Abbreviations

G4: Guanine quadruplexes
qPCR: Quantitative PCR
TERT: Telomerase reverse transcriptase
TO: Thiazole orange
TRAP: Telomere repeat amplification protocol
